# Diagnosis and Management of Fetal Growth Restriction

**DOI:** 10.1155/2011/640715

**Published:** 2011-04-13

**Authors:** Jacqueline E. A. K. Bamfo, Anthony O. Odibo

**Affiliations:** ^1^Obstetrics and Gynecology, London Deanery, London WC1B 5DN, UK; ^2^Division of Maternal-Fetal Medicine and Ultrasound, Department of Obstetrics and Gynecology, School of Medicine, Washington University, Campus Box 8064, 4566 Scott Avenue, St. Louis, MO 63110, USA

## Abstract

Fetal growth restriction (FGR) remains a leading contributor to perinatal mortality and morbidity and metabolic syndrome in later life. Recent advances in ultrasound and Doppler have elucidated several mechanisms in the evolution of the disease. However, consistent classification and characterization regarding the severity of FGR is lacking. There is no cure, and management is reliant on a structured antenatal surveillance program with timely intervention. Hitherto, the time to deliver is an enigma. In this paper, the challenges in the diagnosis and management of FGR are discussed. The biophysical profile, Doppler, biochemical and molecular technologies that may refine management are reviewed. Finally, a model pathway for the clinical management of pregnancies complicated by FGR is presented.

## 1. Introduction

Fetal growth restriction (FGR) refers to a fetus that has failed to achieve its genetically determined growth potential and affects up to 5–10% of pregnancies. Fetal growth restriction is associated with an increase in perinatal mortality and morbidity. This is because of a high incidence of intrauterine fetal demise, intrapartum fetal morbidity, and operative deliveries. In preterm FGR, which occurs before 34 weeks gestation, iatrogenic prematurity is a pertinent issue. Neonates affected by FGR suffer from respiratory difficulties, polycythemia, hypoglycemia, intraventricular hemorrhage, and hypothermia [1–3]. In the long-term cerebral palsy, developmental delay and behavioral dysfunction can occur. Increasing evidence points to a link between FGR and adult metabolic syndrome [4, 5]. Barker et al. found, in a longitudinal study of 13 517 men and women born between 1924 and 1944 in Helsinki University Hospital that a combination of small size at birth, followed by accelerated weight gain during childhood, seemed to be responsible for an increased risk of coronary heart disease, type 2 diabetes, and hypertension. It is postulated that FGR may direct a fetal compensatory mechanism that alters adult susceptibility to disease [4, 5]. 

The occurrence of a fetus that is small for gestational age (SGA) and thus smaller than its expected size is well recognized. The diagnostic challenge is in distinguishing SGA pregnancies from FGR pregnancies because the majority of SGA pregnancies are associated with a good prognosis compared to FGR pregnancies [6]. The World Health Organization defines SGA as a neonatal weight of less than 2500 grams at term [7], a definition that eliminates the impact of accurate pregnancy dating and allows its use in developing countries. The use of an estimated weight below the 10th percentile for gestational age or weight that is less than two SD below the anticipated value for the gestational age [8] is more widely adopted in developed countries. In some cases, SGA is defined as weight below the 5th or even 3rd percentile. Fetuses with a birthweight below the 10th percentile may not be growth restricted but rather constitutionally small. The lower the percentile cut-off, the greater the recognition of FGR [9]. Whilst workable, these definitions risk failing to detect fetuses that have fallen across percentile lines from their original trajectories but remain above the 10th percentile. Growth decline and rate of decline in late pregnancy are important considerations [10]. Marconi et al. recently evaluated the outcome of FGR infants with abnormal pulsatility index of the umbilical artery according to the neonatal birth weight/gestational age standards and intrauterine growth charts. They found that 47% of FGR pregnancies had a birthweight above the 10th percentile (FGR/appropriate for gestational age) compared to 53% with birthweight below the 10th percentile (FGR/SGA). The neonatal morbidity and mortality were similar in FGR of the same clinical severity, whether or not they could be defined as appropriate or small for gestational age according to the neonatal growth standards [11]. Therefore, relying on growth charts and standards, or simple percentile cut-offs, may be insufficient for diagnosing FGR. Mathematical models by Deter et al. and customized growth charts by Gardosi have been shown to improve the detection of growth restriction [12–14]. Deter defined the Prenatal Growth Assessment Score (PGAS) and Neonatal Growth Assessment Score (NGAS) as measures for detecting growth abnormalities. The NGAS allows identification of neonates that had growth problems in utero and reflects an overall assessment of growth outcome. Evaluation of neonatal growth abnormality is important because of the potential postnatal neurological and behavioralal consequences of pathological pregnancies. Deter's model's have proved useful in detecting FGR in multifetal pregnancies [13, 15, 16]. Gardosi's customized charts adjust for maternal factors with the benefit of reducing adverse outcome. There is some evidence that correcting for maternal characteristics alone may not be sufficient to improve detection of FGR [17, 18]. Developments in Doppler of the maternal and fetal circulation, fetal heart rate analysis, and biophysical profile have improved the diagnosis of both FGR and SGA. SGA pregnancies often exhibit normal fetal Doppler [19], whilst FGR due to placental disease exhibits characteristic maternal and fetal Doppler abnormalities. This paper discusses the challenges in the diagnosis and management of FGR.

## 2. Etiology and Pathophysiology

### 2.1. Causes

It is important to identify etiological factors in FGR because this directs the managing physician to an early diagnosis. The causes of FGR are fetal, maternal, environmental, and placental (Table 1). Fetal causes are less common and include aneuploidy (trisomies 13, 18, and 21), fetal malformations, and congenital infections (rubella, cytomegalovirus, rubella, toxoplasmosis) [20]. Multifetal gestations have a high incidence of FGR. About 20–30% of dichorionic twin pregnancies will suffer from FGR, as will 40% of monochorionic twin pregnancies. This is due to placental sharing which places a stress on the uteroplacental circulation. Maternal conditions such as pre-eclampsia and pre-existing or gestational hypertension are leading causes of maternal mortality and morbidity, and are complicated by FGR in 30 to 40% of cases [21]. Diabetes is complicated by FGR in 10 to 20% of cases irrespective of glycemic control [22]. Maternal vascular disease and thrombophilia can lead to uteroplacental hypoperfusion thereby impairing fetal growth [23]. Hypoxia secondary to cardiac, respiratory and hematological disorders may cause FGR. Similarly, tobacco and toxic drugs will compromise the intrauterine environment [24–30]. These disorders destroy the placental through chronic infarctions and abruptions, villitis, or fetal thrombotic vasculopathy [24–30]. In over 20% of fetuses with idiopathic FGR, confined placental mosaicism (the presence of at least two cell lines with different chromosomal complements) exist in the placenta [31].

### 2.2. Placental Dysfunction in Fetal Growth Restriction

Normal placental development and functional integrity are essential for normal fetal growth. There is extensive evidence demonstrating that placentation is inadequate in pregnancies complicated by FGR [32, 33]. The trophoblast is a metabolically active tissue, producing hormones, absorbing nutrients and eliminating unwanted waste. In early normal pregnancy, the trophoblast invades the maternal spiral arteries with loss of smooth muscle and elastic lamina from the vessel walls as far as the inner third of the myometrium, leading to a 5–10-fold dilation at the vessels and establishment of the uteroplacental circulation. Using dimensions in the literature derived from three-dimensional reconstructions, Burton and colleagues modeled the effects of terminal dilation on inflow of blood into the placental intervillous space at term and observed a modest impact of dilation on blood flow. They found that dilation slows the rate of flow from 2 to 3 m/s in the nondilated part of an artery of 0.4–0.5 mm diameter to approximately 10 cm/s at the 2.5 mm diameter mouth, depending on the exact radius and viscosity. In the absence of conversion, blood will enter the intervillous space as a turbulent jet at rates of 1-2 m/s, which could damage villous architecture, rupturing anchoring villi and creating echogenic cystic lesions as evidenced by ultrasound [34]. Histological features seen in the placenta of pregnancies complicated by FGR include damage to branching angiogenesis with long unbranched intermediate and terminal villi, cytotrophoblast proliferation, trophoblast apoptosis, fibrin deposition, syncytial knotting and bridging, and enhanced villous maturation [30, 35].

## 3. The Diagnosis of FGR

The maternal history allows individual risk factors for FGR to be ascertained. Socioeconomic concerns present opportunities for primary prevention. High-risk populations include women with poor lifestyle choices, low prepregnancy weight, or weight gain during pregnancy, previous preterm delivery, and previous pregnancy affected by FGR [20, 36–38]. Pregnancy counseling allows the likelihood of FGR recurrence to be discussed [20, 38]. In maternal medical conditions, recurrence risk is high and disease control is a priority. Where there has been a previous stillbirth, it is essential to establish the cause because of the high incidence of FGR in such cases.

### 3.1. Clinical Diagnosis of FGR

A prerequisite for a judicious diagnosis of FGR is accurate dating of the pregnancy. The last menstrual period, when certain, reliably dates the pregnancy. Alternatively, dating is performed with sonography [39]. Abnormal fetal growth is detected with the clinical suspicion of a subnormal uterine size, followed by abdominal palpation and direct measurement of the symphyseal-fundal distance [40]. Abdominal palpation has a sensitivity of 30% for detecting SGA fetuses. The symphysis-fundal distance has a sensitivity of 27–86% and specificity of 80–93% for detecting SGA [41]. The use of customized symphysial-fundal distance charts which take into account anthropometric characteristics and ethnicity reportedly improve detection [42].

### 3.2. Role of Sonography in the Diagnosis of FGR

Ultrasound is the benchmark for accurate pregnancy dating and diagnosis of FGR. However, there is room for error and FGR is undetected in about 30% of routinely scanned cases and incorrectly detected in 50% of cases [43]. In the 1970s, Robinson and Fleming described pregnancy dating according to the fetal crown-rump length (CRL). A first trimester CRL that is within 5 days of the patient's menstrual dating will accurately date the pregnancy. Transvaginal ultrasound can visualize a gestational sac as early as 4.5 weeks. The gestational sac grows at a mean diameter of 1 mm per day and can be used to correctly determine gestational age [44, 45]. Some authors have shown that it is possible to detect first trimester FGR. In a retrospective review of 4229 pregnancies, Smith and colleagues found that a first-trimester CRL that was two to six days smaller than expected was associated with an increased risk of a birth weight below 2500 g (relative risk, 1.8), a birth weight below 2500 g at term (relative risk, 2.3), a birth weight below the fifth percentile for gestational age (relative risk, 3.0) and delivery between 24 and 32 weeks of gestation (relative risk, 2.1) [46]. Mook-Kanamori et al. has found that the association between first trimester FGR (defined as gestational age-adjusted CRL in the lowest 20% of the population) and low birth weight was independent of maternal physical characteristics and lifestyle choices [47]. Furthermore, a significant relationship between biparietal diameter (BPD) growth rate between the first and second trimesters and adverse pregnancy outcome has been reported. Fetuses with growth rates less than 2.5th centile had an OR of 4.79 (95% CI, 1.43–15.99) for perinatal death and an OR of 2.64 (95% CI, 1.51–4.62) for birth weight less than the sonographically estimated mean fetal weight (adjusted for gestational age) −2 SD [48].

Second trimester pregnancy dating (prior to 20 weeks gestation) is reliable if the fetal biometry is within 10 days of the menstrual date [45]. Femur length, head circumference, BPD, and abdominal circumference are used up to 28 weeks gestation [49]. Beyond 20 weeks, the BPD may diverge from the correct gestational age by 12–15 days, extending to 21 days after 30–32 weeks gestation. Because of this discrepancy, femur length is frequently chosen as a dependable indicator of gestational age in late pregnancy [50, 51]. Estimated fetal weight is calculated using polynomial equations combing BPD, femur length, and the abdominal circumference. The most common formulas are those reported by Shepard et al. and Hadlock et al. [52, 53]. Using these formulas, FGR is typically defined as estimated fetal weight less than the 10th, 5th, or 3rd percentile for the gestational age or below 2 standard deviations of the mean for the gestational age [54, 55].

Traditionally, standards for birthweight for gestation have been based on the average of the population. However, fetal growth is complex especially in the third trimester of pregnancy. There remains a need for a method that distinguishes fetuses that have failed to maintain their growth potential close to delivery and those that are normal or genetically small. Deter has suggested an individualized fetal growth evaluation method where each fetus serves as its own control [16]. This method has effectively identified growth abnormalities in fetuses and neonates except in prenatal cases where only a change in soft tissue has occurred. Individualized growth assessment (IGA) observes changes in sonographic measures over time points. A model, developed by Rossavik calculates expected growth trajectories in the late 2nd trimester and 3rd trimester for an individual fetus (each fetus being its own control) and then compares actual and expected third trimester growth by calculation of Percent Deviation and Growth Potential Realization Index values. Multiple sets of anatomical parameters (Prenatal Growth Profile and the Neonatal Growth Profile) are used in the models. The calculated growth trajectories of head circumference (HC), abdominal circumference (AC), thigh circumference (ThC), femur diaphysis length (FDL), crown-heel length, and estimated weight (EWT) are incorporated in the models [16, 56]. Prenatal Growth Assessment Scores and Neonatal Growth Assessment Scores (NGASI) are calculated from Percent Deviation and Growth Potential Realization Index (GPRI) values for the anatomical parameters, respectively [16, 56]. A modified Neonatal Growth Assessment Score (mNGAS) allows for individual growth variability in growth abnormalities, and has been shown to accurately detect growth restricted neonates [15]. 

Gardosi has proposed standards according to an individual growth potential calculated for each baby in each pregnancy. Gardosi's customized standards are based on certain principles. Firstly, the standards are adjusted or customized for sex as well as maternal characteristics such as height, weight, parity, and ethnic origin. The standards exclude pathological conditions such as diabetes and smoking which are known to affect birth weight. Prematurity is also excluded due to its association with FGR. Factoring the multiple variables produce, the individually “customized” optimal weight value for each pregnancy (“Term Optimal Weight” (TOW) at 280 days) [14]. These customized birthweight standards have been found to be superior to population-based standards in their associations with adverse outcomes such as antepartum haemorrhage, pre-eclampsia, abnormal Doppler, or Caesarean section for fetal distress, low 5-min Apgar score, need for neonatal resuscitation, neonatal care unit admission, poor neurological outcome, and perinatal death [42, 55, 57, 58]. The Royal College of Obstetricians and Gynaecologists of the United Kingdom have recommended the use of customized ultrasound charts [59]. Whilst there are clear benefits for customized weight centiles [14], recent literature attributes the observed benefits in adverse outcomes to the incorporation of intrauterine-based reference values at preterm ages rather than the adjustment made for maternal characteristics. Hutcheon and colleagues hypothesize that methological differences between the calculation of customized birthweight standards and conventional birthweight standards could lead to these benefits. Whilst maternal factors are adjusted for, the reference values in customized standards at gestational ages other than 280 days are derived from an intrauterine standard (because they use a proportionality formula derived from Hadlock's intrauterine standard to extrapolate 280-day expected weights back to younger gestational ages), as opposed to conventional charts, which are based on birthweights of newborn infants [17, 60]. In a population-based cohort study of 782 303 births, Hutcheon and colleagues found that relative risks of stillbirth and early neonatal mortality among SGA births as classified by the intrauterine standard (noncustomized) were similar to those among SGA births as classified by the customized standard and much higher than those among SGA births as classified by the birthweight standard. The use of the customized birthweight standard showed no advantage over noncustomized intrauterine weight standard in predicting perinatal mortality [18]. Notably, customization for maternal factors did not hinder the identification of high-risk infants. Further studies are needed to corroborate these result. 

This further emphasizes the blurred border between physiological and pathological influences on fetal growth. Bukowski et al. demonstrated that individualized optimal fetal growth norms (physiologic factors were used to individually predict optimal growth trajectory and its variation and growth potential for each fetus) better identify normal and abnormal outcomes of pregnancy compared to existing methods. Growth potential norms correctly classified more pregnancies than population, ultrasound, or customized norms in complicated pregnancies [61]. 

Although largely of historical value, two distinct patterns of FGR have been described by Campbell and Thoms and are briefly mentioned for completeness [62]. In symmetrical FGR, all fetal parts have the same degree of growth. Aneuploidy, congenital infection, and fetal alcohol syndrome are associated with symmetrical FGR. In asymmetrical FGR, the head circumference is spared and the abdominal circumference is reduced. This is attributed to the preferential shunting of blood to vital organs (brain, heart, and adrenal glands) due to uteroplacental insufficiency. The etiology and manifestation of symmetric and asymmetric FGR largely overlap limiting the usefulness of this classification in defining FGR [54]. The ratio of head circumference to abdominal circumference as means of distinguishing asymmetrical growth restriction from symmetrical growth restriction has gained popularity in some fetal medicine units. However, these definitions have been superseded by Doppler and fetal heart rate analysis.

A salient feature observed in FGR is the reduction of amniotic fluid volume. Amniotic fluid volume is estimated by a simple subjective assessment or calculation of the amniotic fluid index (AFI) [63]. Normal AFI implies normal placental perfusion and points to alternative etiologies of FGR such as infection, where AFI may be normal or increased. In contrast, low AFI (oligohydramnios, defined as AFI less than 5 cm or less than the 5th percentile) with intact membranes is most commonly associated with uteroplacental insufficiency. Ultrasound is the most common method of measuring amniotic fluid. One second trimester study compared ultrasound to direct measurement of amniotic fluid by dye dilution techniques, reporting good correlation between the two methods (*r* = 0.815; *P* less than  .001) [64]. Because ultrasound is subjective, there may be inaccuracies. Another study comparing ultrasound to dye dilution techniques in the third trimester found that amniotic fluid index overestimated the actual amniotic fluid volume by as much as 88.7% at lower volumes, and underestimated the actual volume by as much as 53.9% at higher volumes [65]. Reported regression slopes (*r* values) for amniotic fluid index and two-diameter amniotic fluid pocket compared to actual amniotic fluid volume are 0.34 and 0.23, respectively [66]. Clearly, technical competence is important in the measurement of amniotic fluid index.

## 4. Screening for Fetal Growth Restriction

### 4.1. Serum Biochemistry

First trimester combined screening for aneuploidy has been the focus of extensive research. Models incorporate maternal characteristics and serum biochemical markers with nuchal translucency in order to predict adverse outcomes. The levels of some biochemical markers are altered in SGA and FGR pregnancies. A raised maternal serum alpha-fetoprotein (AFP) is associated with an increased risk of low birth weight in the absence of structural abnormality or aneuploidy [67]. Low levels of maternal serum pregnancy-associated plasma protein A (PAPP-A) (at the lowest 5th percentile) are associated with an increased risk of an SGA infant [68, 69]. A recent multicenter study related levels of first trimester PAPP-A and second trimester AFP to adverse pregnancy outcome. In that study, the odds ratio for delivering an SGA infant for women with a high AFP was 0.9 (95% CI 0.5–1.6) and for women with a low PAPP-A was 2.8 (95% CI 2.0–4.0). However, when a low PAPP-A at 10 to 14 weeks gestation and high AFP between 15 and 21 weeks gestation were combined, the odds ratio for delivering an SGA infant was 8.5 (95% CI 3.6–20.0). Thirty-two percent of women with this combination delivered a low birth weight neonate (less than 2,500 g) [70]. Several other placental markers are the subject of continued research including human chorionic gonadotrophin (hCG), ADAM12 (A Disintegrin and Metalloprotease), Placental protein 13 (PP13), serum soluble Fas (sFas) and placental growth factor (PlGF), amongst others. However, results show that detection rates are below levels warranted for large population screening [71–76].

In pre-eclampsia, combinations of biochemical and ultrasonographic markers have been shown to improve the early prediction of preeclampsia. In low-risk populations, combinations including placental protein 13 (PP13), pregnancy-associated plasma protein A (PAPP-A), a disintegrin and metalloprotease-12 (ADAM12), activin A, or inhibin A, measured in first or early second trimester and uterine artery Doppler in second trimester, reveal sensitivities of 60%–80% and specificities >80%. In high-risk populations, the combination of PP13 and pulsatility index in first trimester showed 90% sensitivity and 90% specificity in a single study limited to severe pre-eclampsia [77]. Large studies will be needed to evaluate the full potential of evaluating combining multiple markers and ultrasound in screening for pre-eclampsia and FGR.

### 4.2. Uterine Artery Doppler

Since the 1980s, great strides have been made in the use of uterine artery Doppler in obstetric practice, particularly in the detection of maternal perfusion abnormalities in pre-eclampsia and fetal growth restriction [78–80]. The remodeling of the maternal spiral arterioles during trophoblastic invasion is thought to be mirrored by a fall in impedance to uterine blood flow from the first to the second trimester of pregnancy. This technique measures the resistance to blood flow in the uterine artery (resistance index (RI) or pulsatility index (PI)) by isonating the vessel at its apparent cross over with the external iliac artery [81, 82]. Because trophoblastic invasion was thought to be completed by the second trimester, many of the earlier studies were performed between 20 and 24 weeks gestation. Some authors now believe that trophoblastic invasion peaks in the first trimester and argue that this is a more appropriate time to screen for FGR and pre-eclampsia [83]. Martin et al. reported that increased uterine Doppler PI at 11 to 14 weeks had a sensitivity of 11.7% for detecting birth weight less than the 10th percentile, and 27.8% for FGR requiring delivery by 32 weeks gestation [84]. Others have reported sensitivities of 24% and 16% [85, 86]. Despite these low sensitivities, women with a high uterine artery mean RI (greater or equal to the 75th percentile) are 5.5 times more likely to have an FGR pregnancy [86]. In the second trimester, a multicentre study of about 8000 women, using a PI above the 95th percentile (1.63) reported higher sensitivities if FGR was defined by the 5th compared to the 10th percentile (19% versus 16%) [87]. In FGR requiring delivery prior to 32 or 34 weeks, sensitivities of 56% and 70% have been reported, respectively [80, 87]. Newer studies have attempted to add maternal factors and serum biochemistry such as PAPPA in order to increase detection rates. Unfortunately, sensitivities remain unremarkable [71, 88]. There are also technical concerns with reproducibility, especially in the first trimester. The role of uterine artery Doppler in established FGR and in the third trimester is limited. Doppler assessment of the uterine artery is a work in progress and more studies are warranted to define its role in screening for FGR.

### 4.3. Antepartum Fetal Monitoring

The primary objective of antepartum fetal surveillance is to decrease perinatal mortality and morbidity. When FGR is suspected or identified, monitoring tools aim to detect features of fetal acidosis-hypoxemia, which could lead to permanent fetal neurological damage or stillbirth. Nonreassuring tests prompt delivery, whilst reassuring tests delay delivery and minimize iatrogenic prematurity especially at thresholds' of viability. Fetal heart rate monitoring, biophysical profile scoring, and multivessel Doppler are commonly used tests.

### 4.4. Fetal Heart Rate Monitoring

Fetal heart rate analysis or nonstress test is a widely used tool in monitoring high-risk pregnancies. It aims to determine fetal well-being by assessing the fetal heart rate baseline, variability, and periodic changes. A normal reactive test is likely to reflect adequate oxygenation of the fetal central nervous system. Because the fetal heart rate monitoring is interpreted using visual inspection, it is prone to a significant intraobserver and inter-observer variation and therefore has a high false positive rate for abnormality. In premature fetuses, particularly those with FGR, interpretation is challenging [89]. Furthermore, in at least 50% of fetuses with a nonreassuring analysis, the neonate is found not to have evidence of acidosis [90]. Computerized fetal heart rate analysis has been introduced to reduce inconsistencies in the interpretation [91]. Computerized fetal heart rate analysis refines the interpretation of parameters and allows determination of short-term and long-term variation, which are frequently reduced in fetal academia [92, 93]. The contraction stress test is another surveillance tool. Whilst largely superseded by fetal heart rate analysis and multivessel Doppler, it remains useful in uncovering a dysfunctional placenta. During this test, the infusion of oxytocin causes uterine contractions, which are associated with diminished placental perfusion pressure. Fetuses with diminished oxygen reserve will manifest late deceleration on the fetal heart rate analysis. Freeman et al. reported that a corrected perinatal death rate was 176.5/1000 for patients with positive CST compared with 2.3/1000 for patients with a negative test [94].

### 4.5. Biophysical Profile

The fetal biophysical profile (BPP) is a group of measurements that includes the amniotic fluid volume, fetal tone, fetal movements, fetal breathing movements, and fetal heart rate monitoring (NST). When normal, each parameter receives two points, for a maximum total of ten points. In the USA, it is the most acceptable method of noncontinuous fetal well-being assessment [95–97]. The individual components of the BPP all reflect fetal well-being. If evaluation of the AFI reveals oligohydramnios, this calls for further evaluation irrespective of the overall score. The measurement of amniotic fluid volume serves as an indirect estimate of fetal urine production, a marker of fetal renal perfusion. When all other parameters are within normal limits, the need for an NST is questionable. Manning et al. described a high-risk pregnancy protocol where routine NST was not performed when all other BPP parameters were normal [98]. Others view the NST and the BPP as independent predictors of normal outcome [99]. The BPP is usually performed to lower the false positive rate of the NST; however, the BPP has a false positive rate ranging from 75% for a score of six to 20% for a score of zero. Vibroacoustic stimulation, a noninvasive technique that stimulates fetal activity during the BPP test, has been suggested as a means of reducing the false positive rate [100]. The main advantages of the BPP test are the direct assessment of fetal behavior and the technical ease in performing the test. The disadvantages are the performance time required (at least 30 minutes), the dependence on visual interpretation of the NST, and the indirect provision of information regarding fetal cardiovascular status and perfusion. Furthermore, randomized trials comparing the BPP with other tests are lacking [101].

### 4.6. Doppler Velocimetry of Blood Flow

**Figure 1:**
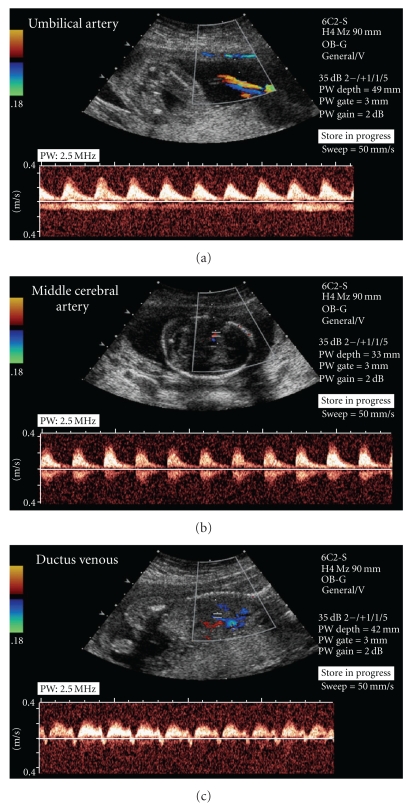
Doppler velocimetry of fetal vessels. (a) depicts a normal umbilical artery flow waveform. (b) depicts a normal waveform of the middle cerebral artery, and (c) depicts an abnormal ductus venosus waveform, showing a reversed “a” wave.

Evaluation of placental and fetal Doppler blood flow has significantly altered the management of FGR. Doppler is used to determine vascular resistance and end organ function. Doppler assessment of the umbilical artery (UA, Figure 1(a)) evaluates blood flow from the fetus to the placenta and therefore reflects placental vessel resistance. Doppler assessment of the middle cerebral artery (MCA, Figure 1(b)) reflects fetal cerebral blood flow. Doppler interrogation of the ductus venosus (DV, Figure 1(c)) detects alterations in fetal cardiac function. The DV is often abnormal in severe cases of FGR [102, 103]. 

The UA is the most commonly used Doppler surveillance tool in women diagnosed with FGR. The usefulness of UA Doppler lies in its ability to distinguish FGR caused by placental disease from nonhypoxic or constitutional causes of SGA pregnancies. Early in placental dysfunction, there is an increase in the resistance to blood flow through the UA, seen as increased systolic/diastolic (S/D) flow ratio or PI. In severe placental insufficiency, diastolic flow becomes absent or reversed, a finding associated with increased perinatal mortality and morbidity [104–106]. 

Monitoring FGR pregnancies using UA Doppler has been shown to reduce mortality rate (OR 0.67, 95% CI, 0.47–0.97) and lower the need for antepartum admissions, labor induction, and Caesarean deliveries [106, 107]. 

The growth restricted fetus reorganizes its blood flow, shunting blood from visceral, and less essential organs, to vital organs such as the brain, heart and adrenal glands. This “brain-sparing” effect is detected by Doppler flow assessment of the MCA, evidenced as decreased systolic/diastolic ratios or PI (Figure 1(b)). In cases of severe fetal hypoxia, there is a rebound increase in S/D ratio and diminished perfusion even to the brain. The MCA Doppler does not consistently predict fetal deterioration [19, 108]. The combination of elevated MCA PI with UA Doppler may have a role in optimizing the timing of the delivery of the growth restricted fetus [107, 109, 110]. Interestingly, MCA peak systolic velocity (used in the management of fetal anemia) shows promise in the follow up of fetuses with an established diagnosis of FGR [111]. 

The fetal veins that are currently evaluated by Doppler include the DV, inferior and superior vena cava, and the umbilical vein. Changes in blood flow in these vessels reflect the compliance of the fetal heart and its ability to cope with preload. A decreased or reversed “a” wave of the DV (Figure 1(c)) is evidence of decreased forward flow in atrial systole, while pulsations in the umbilical vein reflect increased central venous pressure [112]. The ductus venosus is a unique vessel and only a few studies have evaluated its haemodynamics. Bellotti et al. compared the ductal ultrasonographic and Doppler findings in two growth-restricted human fetuses with the results of mathematical simulations of ductal dilatation. They observed apparent active DV dilatation. Prolonged ultrasonographic analysis (45 min) showed rapid and substantial changes (>80%) of ductal diameters suggesting a compensatory effect for which a higher proportion of the umbilical flow is shunted through the ductus to the brain and the myocardium [113]. Tchirikov et al. studied the ability of Doppler ultrasound to evaluate ductus venosus blood flow during acute hypoxemia in fetal lambs. Pulsed wave Doppler ultrasound tended to reduce the mean velocity (*V*mean) and the minimum velocity (*V*min) (based on the maximum velocity envelope curve) in the DV, descending aorta, and inferior vena cava. The pulsatility index of the umbilical artery significantly increased at the end of hypoxemia [114]. Kiserud et al. observed that DV shunting was higher and the umbilical blood flow to the liver was less in fetuses with FGR particularly in those with the most severe umbilical hemodynamic compromise. Fetuses with FGR and normal UA Doppler did not shunt significantly more than the reference fetuses but those with raised UA pulsatility index (>97.5(th) percentile) and those with absent/reversed end diastolic flow shunted significantly more. With more DV shunting, these fetuses distributed correspondingly less umbilical blood to the liver, one of the mechanisms being a lower perfusion pressure as reflected in the lower DV blood velocity [115]. In severe FGR, Doppler examination of blood flow volume revealed a significant increase (>90th percentile of control fetuses) in the shunting of umbilical vein blood flow through the DV that was associated with the dilation of the ductal isthmic diameter [116]. These studies suggest that the decrease of the a-wave is determined by a dilatation of the DV.

There have been a number of studies that have evaluated the sequence of appearance of abnormal Doppler parameters in FGR fetuses with placental dysfunction. Hecker et al. described the temporal sequence of changes in fetal monitoring variables in FGR in a prospective longitudinal observational multicenter study of FGR fetuses after 24 weeks of gestation. Amniotic fluid index and umbilical artery pulsatility index were the first variables to become abnormal, followed by the middle cerebral artery, aorta, short-term variation, DV, and inferior vena cava. Perinatal mortality was significantly higher if short-term variation and DV pulsatility index were abnormal compared to only one or neither being abnormal. They concluded that DV pulsatility index and short-term variation of fetal heart rate were important indicators for the optimal timing of delivery before 32 weeks gestation [117]. Baschat et al. tested the hypothesis that hemodynamic changes depicted by Doppler preceded deteriorating biophysical profile score in severe FGR in a longitudinal study of FGR fetuses in the late second and third trimester. Forty-four of 236 intrauterine growth-restricted fetuses (18.6%) required delivery for abnormal biophysical profile scoring. Three principal patterns of Doppler deterioration were observed: (i) worsening umbilical artery PI, advent of brain sparing, and venous deterioration (72.7%); (ii) abnormal precordial venous flows, advent of brain sparing (13.6%), and (iii) abnormal ductus venosus only (9.1%). In the majority (70.5%), Doppler deterioration was complete 24 h before biophysical profile score decline. In the remainder (25%), Doppler deterioration and biophysical profile score <6/10 were simultaneous [102]. Ferrazzi et al. conducted an observational study in a tertiary care/teaching hospital on FGR fetuses before 32 weeks' gestation to compare Doppler changes as a function of time. Delivery was based on a nonreactive fetal heart rate tracing and not on Doppler information. Firstly, for each vessel there was a progressive increase in the percent of fetuses developing a Doppler abnormality. Secondly, severely FGR fetuses followed a progressive sequence of acquiring Doppler abnormalities, which were categorized into “early” and “late” Doppler changes. Early changes occurred in peripheral vessels (umbilical and middle cerebral arteries; 50% of patients affected 15-16 days prior to delivery). Late changes included umbilical artery reverse flow, and abnormal changes in the DV aortic and pulmonary outflow tracts (50% of patients affected 4-5 days prior to delivery). The time interval between the occurrence of early and late changes was significantly different. Late changes in vascular adaptation by the severely growth-restricted fetus are the best predictor of perinatal death [118].

Presently, it is unclear whether or not there is a reproducible “sequence” of venous flow alterations in the growth-restricted fetus to be applied outside of specialist fetal medicine units. At times, the observed changes in the umbilical vein and ductus venosus temporarily appear to overlap. Arterial and venous Doppler indices may independently predict fetal deterioration. In the majority of severely FGR fetuses, sequential deterioration of arterial and venous flows precedes terminal changes. Integration of serial Doppler evaluation of the umbilical artery, middle cerebral artery and DV to FGR surveillance may improve prediction of outcome and the timing of intervention [112, 119, 120]. 

Myocardial performance index is a new parameter that may be useful in fetal monitoring. 

A recent study has explored the sequence of changes of Myocardial Performance Index (MPI), 

Aortic Isthmus (AoI), and Ductus Venosus (DV) in fetuses with early-onset FGR. 

Myocardial Perfor- mance Index, AoI PI, and DV PI increased with progressive fetal 

deterioration; however, they crossed the 95th percentile at 26 days, 12 days, and 5 

days before delivery, respectively. At the last examination before delivery, 

the proportion of increased MPI (70.4%) was significantly higher than that of abnormal 

AoI PI (55.7%) and DV PI (47.8%) [121]. Further research is needed to assess the potential 

of MPI.

### 4.7. Histopathological and Molecular Diagnostics

Technological advances in the use of molecular probes have enabled us to evaluate disease mechanisms and develop laboratory-based assessment of placental injury. Genomic, proteomics, and metabolomic tools may refine disease diagnosis and provide therapeutic targets in FGR pregnancies. Acquisition of fetoplacental tissues or cells will be necessary. Currently, sampling of amniotic fluid, fetal blood, maternal blood, and tfetoplacental or transabdominally obtained placental tissues is possible. The patterns of gene expression that define hypoxic injury to trophoblasts are not well understood. However, studies using techniques such as high-density oligonucleotide microarrays, in situ hybridization, and quantitative PCR are reporting that placental villi from human pregnancies complicated by FGR demonstrate characteristic changes in “hypoxic trophoblast signature transcripts” [122]. For example, upregulation of transcripts for vascular endothelial growth factor, connective tissue growth factor, follistatin-related protein, N-Myc downstream-regulated gene1, and adipophilin (ADRP), and downregulation of human placental lactogen and PHLDA2 [122] have been shown. For instance, dysregulation of transcripts like CRH, IGF1, IGF2, AGTR1, leptin, and sFlt have also been described. Imprinted genes such as the maternally expressed/paternally repressed Phlda2 or the paternally expressed/maternally repressed gene Mest are differentially expressed in placentas from FGR fetuses [123]. These genes regulate fetal and placental growth, with paternally expressed genes encouraging growth and maternally expressed genes reducing growth [124]. Protein families such as cytokines, growth factors, and angiogenic peptides are suggested to play a role in the pathogenesis of FGR [125]. Proteomic techniques may provide an adjunct to the genomic approaches. These techniques are novel; however, the potential combination of fetal biophysical testing and informatics-based molecular analysis may prove useful in the future management of FGR.

## 5. Clinical Management of FGR

### 5.1. Therapeutic Interventions to Abolish or Attenuate the Impact of FGR

The initial management of FGR entails eliminating proven causes of impaired growth and encouraging a healthy intrauterine environment. Measures such as improved nutrition, smoking cessation, avoidance of drugs, and control of maternal disorders including hypertension and renal dysfunction are important. When present, infectious diseases should be treated. Sonography is vital to identify fetal malformations particularly if lethal and offer fetal karyotyping. Previous studies including meta-analysis evaluating interventions such as plasma volume expansion, oxygen supplementation, administration of glucose or amino acids, and administration of low-dose aspirin to the mother did not show a significant impact on perinatal outcomes [126–128]. Smoking cessation and antimalarial therapy appeared to prevent FGR, but were ineffective in established FGR. Notably, a recent Cochrane review suggests that nutritional advice to women and balanced energy/protein supplements may be beneficial in ameliorating the risk of the disease [129, 130].

### 5.2. Surveillance of the Growth-Restricted Fetus

In the past, studies evaluating benefits of antepartum surveillance methods in FGR pregnancies have been limited by wide inclusion criteria of high-risk conditions (FGR, pre-eclampsia, diabetes) and diverse methodology. A recent review of four studies involving 1,588 high or intermediate risk pregnancies reported that antenatal fetal heart rate monitoring NST did not appear to have a significant effect on perinatal mortality or morbidity [131]. Similarly, a Cochrane review on the role of BPP in the management of high-risk pregnancies found no difference between biophysical profile and alternative forms of fetal assessment [101]. In contrast, Manning et al. suggest that NST or BPP may define high-risk pregnancies where the risk of in utero demise is high, and therefore early delivery might be warranted [96]. Recent evidence suggests that UA Doppler may reduce the need for antepartum interventions and lower perinatal morbidity and mortality [106]. Doppler interrogations of MCA, ductus venosus, or other venous vessels are increasingly being used in obstetric practice.

### 5.3. Timing the Delivery of the Growth-Restricted Fetus

Currently there is no single test that dictates the optimal timing of delivery. Once FGR is established, signs of fetal deterioration in biophysical and sonographic indices direct a timed delivery. When FGR is diagnosed at full term (≥37 weeks by reliable dates), delivery is favored. A recent study reported that in late-onset SGA fetuses with normal Doppler velocimetry at diagnosis, there is progression from 37 weeks' gestation with worsening cerebroplacental ratio, followed by a decrease in MCA-PI [132]. There is evidence that term singletons with SGA birth weights have a five to seven-fold risk of developing cerebral palsy compared with gestational age-matched infants with birthweights within normal limits. Hence, there appears to be no advantage in delaying delivery once term is reached [133]. 

 At 34–37 weeks, the incidence of significant neonatal morbidity is low and the risk of hyaline membrane disease can be assessed by amniocentesis, hence, delivery is a less complex issue [134]. When FGR occurs before 34 weeks gestation, decision to deliver is more difficult and is individualized. The recently concluded European Growth Restriction Intervention Trial (GRIT) [135] compared the effect of preterm delivery (at 24–36 weeks), based on UA Doppler waveform, with that of delayed delivery by other clinical indicators. Women were randomly assigned to immediate delivery for abnormal UA Doppler or a delayed delivery based on the managing physicians belief that delivery was warranted, subject to worsening tests or a favorable gestational age. The main outcome variables were survival to hospital discharge and developmental quotient at two years of age. Of 548 pregnancies randomized into the study, there was no difference in overall mortality between the immediate delivery and the delayed delivery groups [135]. In the 2-year follow-up study, there were no significant differences between the groups in death or disability rates [136]. Interestingly, the fact that the managing physicians were prepared to time the delivery even as early as 29–34 weeks using a randomization process points to the uncertainty regarding risk/benefit of immediate delivery. Unfortunately, the GRIT study failed to address specific triggers for timing of delivery. Therefore, the question as to the best indicator for delivery remains unanswered by that study.

The longitudinal studies characterizing the sequence of changes in fetal Doppler's have shown that DV flow waveforms become abnormal only in advanced stages of fetal compromise [118]. Furthermore, DV abnormality precedes the loss of short-term variability in the fetal heart rate, and in 90% of cases it becomes abnormal only 48–72 hours before the biophysical profile [102, 117]. Hence, integration of both DV Doppler and biophysical profile in the management of preterm IUGR seems logical. Baschat et al. provided neonatal outcomes specific for early-onset placenta-based fetal growth restriction quantifying the impact of gestational age, birth weight, and fetal cardiovascular parameters. From 24 to 32 weeks, major morbidity declined (56.6% to 10.5%). FGR neonates delivered after 26 weeks had at least 50% chance of survival. FGR neonates delivered before 26 weeks had less than 50% chance of survival. Birthweight of 600 g, DV Doppler and cord artery pH predicted neonatal mortality and DV Doppler alone predicted intact survival [137].

There is an ongoing randomised multicentre study of timing delivery in women found to have FGR on ultrasound scan at gestations between 26 and 32 weeks (trial of umbilical and fetal flow in Europe, TRUFFLE). It is aimed at evaluating the role of DV assessment (early DV changes (pulsatility index >95th centile) and late DV changes (a-wave reaches the baseline, i.e., 0 cm/s) compared to standard management based on fetal heart rate monitoring (short-term variation below preset cut-offs based on gestation) for timely delivering early-onset IUGR cases. The primary outcome for this trial is survival without neurodevelopmental impairment at 2 years of age corrected for prematurity. http://www.trufflestudy.org/.

**Figure 2:**
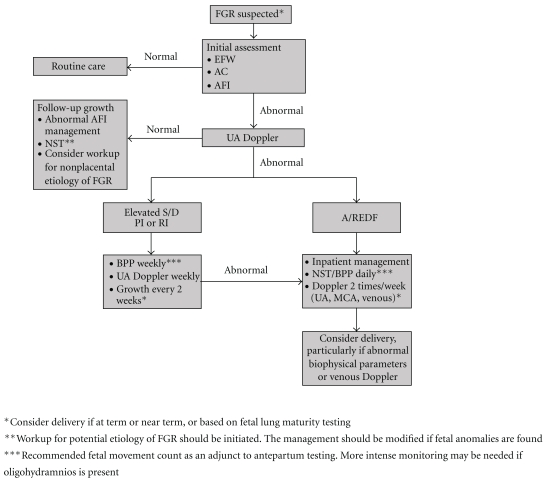
A proposed monitoring scheme in pregnancies complicated by FGR. This figure focuses on FGR associated with placental dysfunction. See text for additional details.

Different obstetric centers currently depend on either biophysical tests (NST, computerized NST, BPP, and alike) or Doppler blood flow studies in order to time delivery. Using published data, we developed a decision tree to explore the optimal antepartum test for timing the delivery of the preterm FGR fetus [138]. This is specific to our unit. Our retrospective decision analysis indicated that BPP was the best test to guide decisions on delivery of the preterm growth-restricted fetus. Clearly, our results must be corroborated by a well-designed, prospective clinical trial prior to universal acceptance.  Figure 2 presents a proposed pathway for the antenatal monitoring of the growth-restricted fetus. The decision to deliver a growth-restricted fetus must be individualized and balanced with available local perinatal-neonatal services. What is clear is that after 34 completed weeks, the appearance of advanced, worsening signs of fetal deterioration, such as UA absent or reversed diastolic flow, persistent nonreassuring NST, a BPP score of ≤4, reversed “a” wave of the DV, or umbilical vein pulsations suggest the need for immediate delivery [139]. Furthermore, the decision to administration of a course of steroids, which reduce the incidence of hyaline membrane disease in every preterm, growth-restricted fetus, is unequivocal.

### 5.4. The Mode of Delivery of the Growth-Restricted Fetus

There is contradictory evidence in the literature regarding the best mode of delivery of the growth-restricted fetus. A vaginal delivery is rarely attempted when biophysical assessment of fetal status is nonreassuring prior to labor because fetal hypoxia may be exacerbated. Even when biophysical parameters are reassuring, clinicians vary in their decisions. In the GRIT study, one third of the pregnancies with FGR were delivered by the abdominal route, yet it is not clear if there was any benefit from this approach [135]. A recent Cochrane review pointed out that Caesarean delivery for SGA fetuses was associated with a lower rate of respiratory distress syndrome, neonatal seizures, and death, but these trends were statistically insignificant [140]. Obviously, other obstetrical factors such as the gestational age, cervical status, fetal presentation, and maternal medical complications may influence the choice of delivery route. Because the growth restricted fetus may require specialized neonatal care, particularly when the delivery occurs preterm, it is prudent to transfer the fetus to a well-equipped center where experienced perinatal-neonatal care is available.

## 6. Conclusion

Significant advances have been made in the understanding of the complex etiology and pathophysiology of FGR. This knowledge will certainly aid the clinician to optimize antepartum monitoring and time delivery of FGR infants. Biophysical tests and multivessel Doppler have predictive abilities in detecting academia but their strengths need further evaluation. The temporal correlation between the commonly used tools and preterminal fetal status remains unclear. There are emerging recommendations to combine the use of multiple monitoring tools in models to improve the prediction of adverse outcome. Nonetheless, there is still no definitive cure for FGR and management strategies for pregnancies complicated by FGR, are based on limited evidence. Well-designed randomized clinical trials are much needed that specifically target the different management options.

## Figures and Tables

**Table 1:** Causes of fetal growth restriction.

*Fetal*	
Aneuploidy (trisomy 13, 18 and 21, triploidy, uniparental disomy)	
Fetal malformations (gastroschisis, omphalocele)	
Multiple gestation	
Infection (toxoplasmosis, rubella, cytomegalovirus, herpes)	

*Maternal*	
Hypertension	
Diabetes	
Renal disease	
Vascular disease	
Inflammatory bowel disease	
Hypoxia (pulmonary disease, cardiac disease)	
Systemic lupus erythematosus, antiphospholipid syndrome	
Thrombophilia (Factor V Leiden heterozygote, Prothrombin gene G20210A heterozygote, MTHFR heterozygote)	
Maternal uterine malformations (myomas, bicornuate, or septate uterus)	
Residing at altitude	

*Placental*	
Placenta praevia	
Placental tumors	
Mosaicism	

*Environment*	
Low socioeconomic status	
Malnutrition	
Smoking	
Alcohol	
Drugs (cocaine, heroin, methadone, cocaine, therapeutic agents)	
